# Functional outcome of prolonged refractory status epilepticus

**DOI:** 10.1186/s13054-015-0914-9

**Published:** 2015-04-30

**Authors:** Alexandre Lai, Hervé D Outin, Julien Jabot, Bruno Mégarbane, Stéphane Gaudry, Rémi Coudroy, Guillaume Louis, Francis Schneider, Nicolas Barbarot, Antoine Roch, Nicolas Lerolle, David Luis, François Fourrier, Anne Renault, Laurent Argaud, Tarek Sharshar, Sébastien Gibot, Pierre-Edouard Bollaert

**Affiliations:** Service de Réanimation médicale, Hôpital Central, CHU de Nancy, Nancy, France; Service de Réanimation médico-chirurgicale, CHI de Poissy-Saint Germain en Laye, Poissy, France; Service de Réanimation Polyvalente, CHU Réunion, Saint Denis de la Réunion, France; Service de Réanimation et de Toxicologie, CHU Lariboisière, Université Paris VI, Paris, France; Service de Réanimation Médico-Chirurgicale, Hôpital Louis Mourier, Colombes, France; Univ Paris Diderot, UMRS 1123, Paris, France; Service de Réanimation Médicale, CHU de Poitiers, Poitiers, France; Service de Réanimation Polyvalente, CHR de Metz-Thionville, Metz, France; Service de Réanimation Médicale, Hôpital de Strasbourg-Hautepierre, Université Louis Pasteur, Strasbourg, France; Service de Réanimation Polyvalente, CH de St Brieuc, St Brieuc, France; Service de Réanimation, Hôpital Nord, CHU de Marseille, Marseille, France; Service de Réanimation Médicale et de Médecine Hyperbare, CHU et Université d’Angers, Angers, France; Service de Réanimation Médico-chirurgicale, Garches, France; Service de Réanimation Polyvalente, Hôpital Roger Salengro, CHU de Lille, Lille, France; Service de Réanimation Médicale, CHU de Brest, Brest, France; Service de Réanimation Médicale, Hôpital Edouard Herriot, Lyon, France; Service de Réanimation Médico-chirurgicale, Hôpital Raymond Poincaré, Garches and Université de Versailles St Quentin en Yvelines, Garches, France

## Abstract

**Introduction:**

To characterize etiology, clinical course and outcomes of patients in prolonged refractory status epilepticus (PRSE) and looking for prognostic factors.

**Methods:**

Retrospective study conducted in patients hospitalized from January 1, 2001 to December 31, 2011 in 19 polyvalent intensive care units in French university and general hospitals. Patients were adults with a generalized convulsive refractory status epilepticus that lasted more than seven days, despite treatment including an anesthetic drug and mechanical ventilation. Patients with anoxic encephalopathy were excluded. Follow-up phone call was used to determine functional outcome using modified Rankin Scale (mRS) with mRS 0–3 defining good and mRS 4–6 poor outcome.

**Results:**

78 patients (35 female) were included. Median age was 57 years. Causes of status epilepticus were various, mainly including prior epilepsy (14.1%), CNS infection (12.8%), and stroke (12.8%). No etiology was found in 27 (34.6%) patients. PRSE was considered controlled in only 53 (67.9%) patients after a median duration of 17 (IQR 12–26) days. The median length of ICU stay was 28 (19–48) days. Forty-one (52.5%) patients died in the ICU, 26 from multiple organ failure, 8 from care withdrawal, 2 from sudden cardiac arrest, 1 from brain death and 4 from unknown causes. PRSE was previously resolved in 20 patients who died in the ICU. At one-year follow-up, there were 12 patients with good outcome and 58 with poor outcome and 8 lost of follow-up. On multivariate analysis, only vasopressor use was a predictor of poor outcome (OR 6.54; 95%CI 1.09-39.29; p = 0.04).

**Conclusion:**

Poor outcome was observed in about 80% of this population of PRSE. Most patients died from systemic complications linked to their ICU stay. Some patients can recover satisfactorily over time though we did not identify any robust factor of good outcome.

## Introduction

Super-refractory status epilepticus (SE) was recently defined as a refractory status epilepticus (RSE) that continues or recurs 24 h or more after the onset of anesthetic therapy [1,2]. It also includes those cases in which SE recurs shortly after the reduction or withdrawal of anesthetic drugs. Others consider the term of late SE when refractory for more than 48 h [3]. Moreover, the term of prolonged refractory status epilepticus (PRSE) has been used for patients with SE that persists or recurs 7 days of more after the onset of continuous general anesthesia [4,5]. About 15% of patients admitted to hospital for SE will become super-refractory [1]. Identified risk factors are head trauma, stroke and central nervous system infections [2]. However, super-refractory SE can occur *de novo* in people with no history of epilepsy and in whom no identifiable etiology can be found, so that it remains a very heterogeneous entity [3]. SE therapy constitutes an emergency for which an early aggressive therapeutic approach is required in order to avoid systemic and neurological complications [3,6,7]. Nevertheless the therapeutic management of prolonged SE remains a *terra incognita* with regard to evidence-based medicine [1,8,9]. The prognosis of RSE has long been considered as poor, including high mortality rates and severe neurological impairment, which may raise ethical concerns on the usefulness of prolonged full treatment in the ICU. However, recent reports suggest that an acceptable recovery could be observed with time in some patients and that the PRSE duration should not be by itself a motive for intensive care withdrawal on behalf of futility [4,5,10]. Here we conducted a multicenter retrospective study to examine the characteristics, etiological and therapeutic features of patients with PRSE according to the above-mentioned definition [4] hospitalized in French ICUs. As in previous reports, we only considered patients who underwent at least 7 days of general anesthesia [4,5]. We also attempted to describe short and long-term prognosis with particular attention to the evolution of the neurological impairment from the ICU discharge.

## Methods

### Study participants

We retrospectively evaluated the data on PRSE treated in the ICU from 1 January 2001 to 31 December 2011. One hundred and thirty French adult ICUs on a list published by the French Language Society of Critical Care Medicine were invited to participate in the study in September 2012. At each participating ICU, one investigator from the medical staff was responsible for collecting the retrospective data. The diagnosis of prolonged PRSE was considered in all patients >18 years old suffering from a generalized convulsive SE, which was considered uncontrolled after general anesthesia (GA) and mechanical ventilation for at least 7 days. Absence of SE control was defined as clinical (rhythmic motor movements) or electrical seizures while under treatment or recurring within 48 h after stopping anesthetic drugs. Patients with complex partial SE and anoxic encephalopathy after cardiac arrest were excluded.

### Data collection

General data included the simplified acute physiological score (SAPS II), the length of ICU stay and common comorbidities often associated with SE. We also recorded the antiepileptic drugs administered from the beginning to the end of the SRSE, the duration of SRSE, the type of neurological monitoring, and the existence of an electrical interruption in the seizure or burst-suppression during the first 7 days. Morbidity and mortality at ICU discharge were also recorded. The degree of disability was estimated with the modified Rankin Scale (mRS) before hospitalization, at ICU discharge, at last news from the patient, and, if available, at one year after ICU discharge [11]. The functional outcome was dichotomized into good (mRS 0 to 3) and poor (mRS 4 to 6) [12]. To obtain this information, local investigators had to contact the attending physician, the patient or the patient’s family.

### Statistical methods

Descriptive results for continuous variables were expressed as mean and standard deviation or as median and interquartile range, depending on the normality of their distribution. Variables were tested for their association with prognosis by using Pearson’s chi-squared test for categorical data and the Mann-Whitney *U*-test for numerical data. A multiple stepwise logistic regression model was established with any covariate with univariate significance of a *P*-value less than 0.10 eligible for inclusion in the model. The model was then further calibrated through Hosmer-Lemeshow testing.

### Ethical considerations

The study was approved by the Ethics Committee of the French Language Society of Critical Care Medicine. According to French law on non-interventional and retrospective studies, patients received information about the study and non-opposition to their participation in the study was sought.

## Results

Among the 130 ICUs invited to participate, 15 reported that no patients fulfilled the inclusion criteria, 19 admitted at least one patient who fulfilled the inclusion criteria in the study period and the remaining ICU provided no information. A total of 78 patients were included. Their baseline characteristics are displayed on Table 1. The etiology of the SE was known or highly suspected in 51 (65.4%) patients and led to a specific treatment when available.

**Table 1:** **Baseline characteristics of patients**

**Patients characteristics**	**Value in 78 patients**
Age, years	57 (36 to 70)
Female	35 (44.8)
History of epilepsy	26 (33.3)
History of alcoholism	27 (34.6)
History of stroke	10 (12.8)
Existing central nervous system pathology	2 (2.5)
History of drug abuse	4 (5.1)
**Etiology of super-refractory status epilepticus**	
Epilepsy	11 (14.1)
Stroke	10 (12.8)
Central nervous system infection	10 (12.8)
Metabolic encephalopathy	5 (6.4)
Neurodegenerative disease	4 (5.1)
Drug abuse	4 (5.1)
Post neurosurgery	4 (5.1)
Inflammatory encephalitis	3 (3.8)
Unknown	27 (34.6)
**Admission modified Rankin scale** ^**a**^	
0	29 (37.1)
1	23 (29.5)
2	13 (16.6)
3	4 (5.2)
4	4 (5.2)
5	2 (2.5)
Simplified acute physiology score II	52.8 ± 14.6

Data are number (%), mean ± SD, or median (IQR). ^a^Data were missing in two patients.

Seventy (89.7%) patients received intravenous benzodiazepines, two received sodium valproate and four underwent GA as their first-line treatment in the attempt to control the seizures. Sixty-five patients (83.4%) had a second-line treatment with anti-epileptic drugs (AED) including fosphenytoin (n = 35), phenobarbital (n = 23), levetiracetam (n = 2), sodium valproate (n = 5) and oxcarbazepine (n = 1) and nine underwent GA. Twenty-seven patients received a third-line AED, including phenobarbital (n = 10), fosphenytoin (n = 10), sodium valproate (n = 5) and levetiracetam (n = 2). During the first week of GA, 65 patients were treated with a median of 2 (1 to 3) other AED, usually through the enteral route. The agents used to induce GA as a first-, second- or third-line treatment were thiopental (n = 58), propofol (n = 17), ketamine (n = 1), halogenated gas (n = 1), combined or switched with continuous midazolam infusion (n = 55) in the first 7 days of SE. After 7 days of unsuccessful treatment, GA was continued using various anesthetic drugs, including thiopental (80%), midazolam (47%), propofol (25%) and in some anecdotal cases, ketogenic diet (n = 7), immunoglobulin infusion (n = 1), vagal stimulation (n = 1), and electroconvulsivotherapy (n = 1). SE was monitored by discontinuous electroencephalogram (EEG) in all but one patient who underwent continuous EEG monitoring. EEG burst suppression was observed in 48 patients (61%) within the first 7 days.

All patients were mechanically ventilated from the time they received general anesthesia. Administration of vasopressors and renal replacement was required in 60 and 14 patients, respectively. Nosocomial infections were observed in 60 patients, 27 of them with at least one episode of ventilator-associated pneumonia. A thrombo-embolic event occurred in five patients. PRSE was considered controlled in 53 (67.9%) patients after a median length of 17 (12 to 26) days of general anesthesia. The median length of ICU stay was 28 (19 to 48) days.

Withholding or withdrawing care was decided in 16 and 8 patients, respectively, after a median stay of 20 (14 to 25) days. Motivations of therapeutic limitation included persistent RSE without improvement or identifiable etiology, stroke with major brain lesions, multiple organ failure, pre-existent chronic alcoholism with neurological and/or hepatic complications, and cerebral tumor without available treatment. Half of the 16 patients who underwent withholding of care survived, only one of them with a good functional outcome at one year (mRS = 2) and all patients who underwent withdrawal of care died. Forty-one (52.5%) patients died in the ICU. Among them, 26 died from multiple organ failure secondary to sepsis, 8 from care withdrawal, 2 from sudden cardiac arrest, 1 from brain death subsequent to severe intracranial hypertension and 4 from unknown causes. PRSE had previously been resolved in 20 patients who died in the ICU.

Surviving patients (47.5%; n = 37) were followed after ICU discharge. Five patients died within one year after ICU discharge. Eight patients were lost to follow up. Twenty-four of the remaining patients had at least one year of follow up. Between ICU discharge and one-year follow up, 13 patients (54.1% of the survivors) had seizures, six of them having a previously known history of epilepsy. However, no recurrence of SE was observed. ICU-acquired paresis was present in 14 patients (58.2% of the survivors).

**Figure 1:**
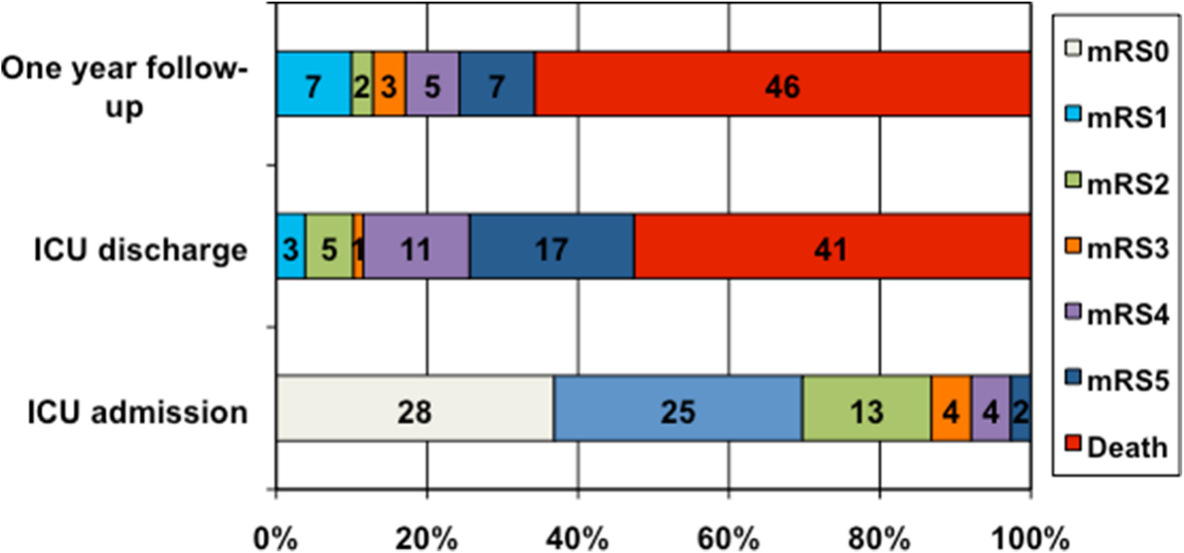
Distributions of scores according to the modified Rankin scale (mRS) on admission, ICU discharge and one-year followup. Data are lacking for two patients on ICU admission; eight patients were lost to follow up at one year.

As shown in Figure 1, there was a marked decline in functional status between admission and ICU discharge with a median increase in mRS of 4 (3 to 5). At one-year follow up, 12 (17.1%) patients had a good outcome with a median mRS decrease of −1 (−2.5 to −1) as compared with ICU discharge mRS.

**Figure 2:**
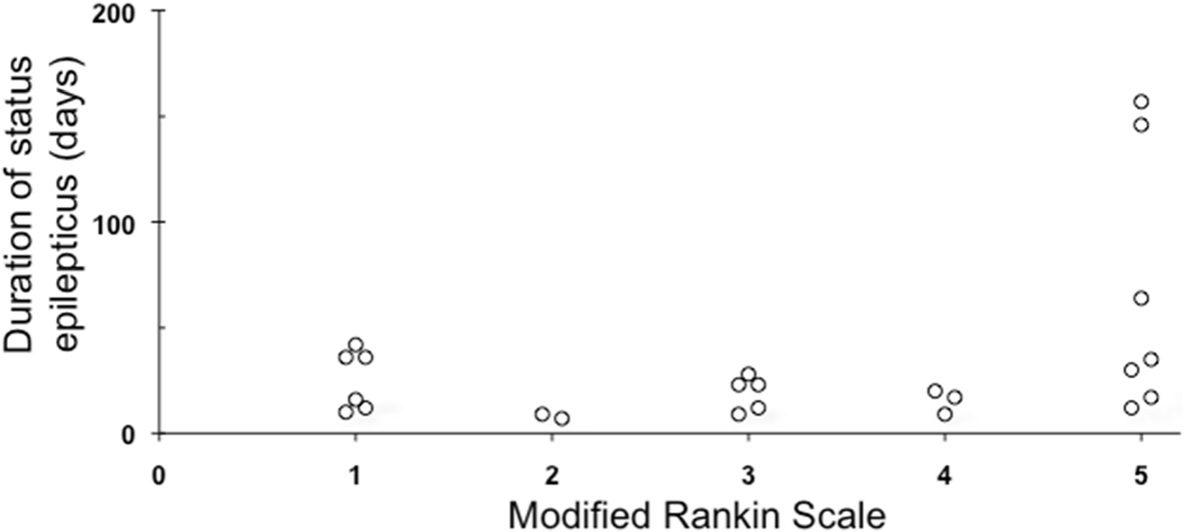
Modified Rankin scale of one-year survivors according to status epilepticus duration.

Univariate analysis (Table 2) showed that male gender, younger age, EEG burst-suppression observed within 7 days, introduction of an etiological treatment and no need for vasopressors were associated with a better one-year outcome. On multivariate analysis, only the use of vasopressors remained significantly predictive of outcome (odds ratio = 6.54; 95% confidence interval 1.09, 39.29; *P* = 0.04). Underlying coexisting illnesses and etiology of PRSE were not related to outcome, except for stroke where all patients (n = 10) had a poor outcome. Drugs used for GA had no statistically significant effect on outcome. The duration of SE did not differ according to outcome. Figure 2 shows that a good outcome is possible after more than 30 days of SE. However, all patients whose SE was unresolved after 60 days (n = 3) and those who stayed more than 90 days in the ICU had a poor outcome (n = 8: extremes 90 to 300 days).

**Table 2:** **Univariate analysis according to one-year outcome**

	**Good outcome (mRS ≤3) n = 12**	**Poor outcome (mRS >3) n = 51** ^**a**^	***P*** **-value**
Male gender	9 (75)	30 (58.8)	0.01
Age, years	42 (31 to 62)]	58 (45 to 71)	0.07
Simplified acute physiology score II	46.9 ± 12.8	53.9 ± 15.4	0.65
Admission mRS	0.5 (0 to 1)	1 (0 to 2)	0.16
Known cause of PRSE	7 (58.3)	31 (60.8)	0.57
Thiopental for general anesthesia	11 (91.7)	47 (92.1)	0.55
EEG burst suppression within 7 days	9 (75)	32 (62.7)	0.054
Status epilpticus duration, days	15 (11 to 37)	17 (11 to 22)	0.95
Length of ICU stay, days	35 (20 to 49)	25 (14 to 45)	0.38
Etiological treatment	8 (66.7)	22 (43.1)	0.0003
Vasopressors	4 (33.3)	32 (62.7)	<0.0001
Nosocomial infections	10 (83.3)	38 (74.5)	0.14
Seizures after ICU discharge	3 (25)	5 (35.7)	0.27
ICU-acquired polyneuropathy	6 (50)	8 (57.1)^b^	0.59

Data are number (%), mean ± SD or median (IQR). ^a^Patients with complete available data; ^b^only in ICU survivors. mRS, modified Rankin scale; PRSE, prolonged refractory status epilepticus; EEG, electroencephalogram.

## Discussion

In this multicenter study, we identified 78 patients with generalized convulsive PRSE from various causes except anoxia over 10 years. All patients underwent at least 7 days of general anesthesia after failure of first-line, and for almost all, second-line anti-epileptic therapies. Overall prognosis was poor, with more than 50% of patients not surviving their ICU stay. Of note, a significant number of patients discharged from hospital with a poor functional status improved thereafter, so that at one-year follow up, nearly half of survivors followed up had good functional outcome. Seeking to identify predictive variables of long-term outcome, we found that systemic complications related to the management of PRSE are probably more relevant than direct SE-related brain damage.

Overall characteristics of the present population did not differ from published series of refractory SE with regard to age and sex ratio in this population excluding children and young adults <18 years old. However, history of epilepsy was found in only one-third of patients, a lower proportion than in cohorts of unselected patients with SE where 47 to 67% rates have been reported [13-15]. Although many different causes of super-refractory SE were observed, no etiology was found in one third of patients. This finding contrasts with other series of super-refractory SE where absence of diagnosis was <20% [4,5,16]. In addition, the relatively low proportion of patients with encephalitis in the present study also differs from other super-refractory SE cohorts where up to 35 to 45% of encephalitis were observed [5,16]. It may be hypothesized that some inflammatory encephalitis where cerebro-spinal fluid and imaging alterations are sometimes absent or very subtle could have been either underdiagnosed or considered only as a hypothetical etiology.

Use of AED drugs was heterogeneous among patients, a finding that was not unexpected in a multicenter retrospective study over ten years. Although American recommendations for the management of status epilepticus were available in 1993 [17] and French recommendations in 1995 then updated in 2009 [7], more precise guidelines about RSE were only recently implemented [18,19]. That could explain the low use of propofol as compared with barbiturates observed in the present study, whereas based on higher mortality rates observed with thiopental or pentobarbital than with propofol in retrospective studies [7,20], the latter anesthetic drug could be preferred [18]. However, use of thiopental had no prognostic value in the present study, as in a recent single-center cohort with RSE reported by Hocker *et al*. [12], so that this question remains controversial.

About 50% of patients did not survive the ICU stay. ICU mortality was a little higher than in previous series of patients with PRSE using the same inclusion criteria, with 43 and 34% of ICU mortality respectively [4,5], but was worse than in RSE where in-hospital mortality rates ranged from 15 to 30% [3,12,21,22]. Of interest, median SE duration was <4 days in two series reporting 16 and 17% RSE mortality respectively [21,22], mean SE duration was 11 days in a series of patients with RSE with 31% mortality [12], and finally, median SE duration ranged from 17 to 19 days in the present study and in the two other studies of PRSE [4,5], which suggests a close correlation between the duration of RSE and ICU mortality. Of note, only one patient died directly from a neurological cause (brain herniation and cerebral death). A majority of patients died from multi-organ failure, in relation to nosocomial infections and sepsis. Although this rate appears higher than in other series of patients with PRSE, infectious complications were also frequently observed while their causative role in precipitating death was unclear [4,12]. Others died from life-supporting treatment withdrawal, often the main cause of death in other series [4,12], which could mask or prevent other causes of death, including sepsis. Almost half of the non-survivors no longer had SE at the time of death. Taken together, causes of death and resolution of SE in most non-survivors suggest that iatrogenic events may play an important role in outcome. That the rate of complications could have been reduced by a larger use of propofol instead of thiopental is possible, but speculative [1,3,16].

Most patients discharged alive had severe functional impairment. However, functional improvement was observed in some patients so that about 50% of the survivors followed up for one year had a good outcome. Surprisingly, seizures were not frequent and no recurrence of SE was observed during follow up as previously observed [5], which suggests that the high resistance of SE to treatment does not predict further drug-resistant epilepsy once controlled.

We sought to identify variables associated with long-term poor outcome. Age was modestly associated with outcome but not in multivariate analysis; a previous meta-analysis of refractory SE found the same association in 193 refractory SE patients [23], but it was not retrieved in more recent cohorts of refractory SE [12,14] or PRSE [5]. Surprisingly, both SE duration and length of ICU stay did not significantly differ between populations with poor or good outcome. However, we also observed that no patient with an SE duration >60 days and/or an ICU stay >90 days had a good outcome. The latter finding is consistent with previous published cohorts of patients with PRSE [5,12]. However, the absence of a statistical link between SE duration and outcome in PRSE does not imply that SE duration is not relevant for prognosis: as exposed above, overall mortality is correlated with median duration of SE, patients with extreme SE duration display a very poor prognosis and SE duration is the determinant of duration of GA. Finally, as also observed in the other cohorts with PRSE [4,5,12], some patients may achieve a good outcome with very prolonged SE duration which could explain the absence of statistical significance of SE duration on long-term outcome within our study and others [4,12]. EEG burst-suppression within 7 days of treatment was weakly associated with a good outcome in univariate but not multivariate analysis: this could be either an indicator of the degree of drug resistance or a marker of a better therapeutic strategy but we are unable to conclude further on that point. The availability of a causative treatment was associated with a good outcome, which is supported by medical common sense, cohort studies [24] and clinical reports where only control of a pathogenic event resolved very prolonged PRSE [25].

Finally, only vasopressor use was found to be associated with poor outcome in multivariate analysis. The high odds ratio was matched with a large confidence interval of limited statistical significance. In a retrospective cohort of 144 episodes of SE, Kowalski *et al*. showed that the need for mechanical ventilation, vasopressors or third-line anesthetic drugs was associated with poor outcome [26]. These covariates were linked together, so that only the use of third-line anesthetic drugs remained significant on multivariate analysis. In the present study, both anesthetic drugs and mechanical ventilation were applied to every patient, which explains their absence of effect on outcome. Vasopressors were always prescribed to correct an arterial hypotension and not to increase cerebral perfusion pressure. While hypotension is common when using thiopental, propofol or even midazolam [23], it could alternatively be subsequent to or potentiated by sepsis, cardio-pulmonary interactions during mechanical ventilation and various other causes. While direct deleterious effects of vasopressors cannot be ruled out [27], the cause of hypotension, including anesthetic drugs, should be considered. Indeed, whatever the cause and mechanisms of vasopressor adverse effects, we have to acknowledge that the strongest prognosis factor we found is more linked to adverse effects of ICU care than SE by itself. In this way, the strong relationship between SE duration and poor prognosis could at least be partially explained by systemic complications of ICU care, especially prolonged general anesthetic drug administration [9,28,29].

About 30% of patients were given a decision to withhold or withdraw life-sustaining therapy. A decision to withdraw life-sustaining therapy was the second cause of death in our series and the first cause in two recent studies in patients with RSE [12] and PRSE [4]. In the present study, most decisions were related to previous severe comorbidities, severe brain damage (stroke, Creutzfeldt-Jakob disease, brain tumor) or multiple organ failure. All patients in whom life-support care was withdrawn (mechanical ventilation, vasopressors) died shortly after withdrawal of care, whereas patients in whom only withholding therapy (no cardiac arrest resuscitation, no therapeutic escalation) was decided survived. Although survival of ICU patients who were given a decision to limit treatment appears surprising, their long-term outcome was very poor (mRS = 5 or death) except for one patient with a mRS of 2. This patient died 6 months later from hepatic cirrhosis (one of the motivations for withholding care).

We are aware that this study has several limitations. It was a retrospective study of a heterogeneous cohort with regard to drug therapy. A selection bias cannot be ruled out with regard to the absence of contribution from the majority of invited ICUs. As in most multicenter studies, management varied by center and the retrospective nature of the study did not allow us to record all pertinent data. The etiology of PRSE was diverse, which did not allow us to identify some causes of PRSE associated with good outcomes. In addition, both the absence of monitoring of SE using continuous EEG and suboptimal achievement of EEG burst-supression or suppression-pattern goals are no longer in agreement with the most recent recommendations [18,19] We assessed outcome using the mRS, which is a recognized scale of autonomy, but it does not inform about quality of life. Finally, the multivariate analysis would have been more robust with a larger population. However, to our knowledge, this is currently the largest available multicenter series of patients with prolonged PRSE.

## Conclusions

This multicenter study confirms that prolonged PRSE leads to high morbidity and mortality rates with about 80% of patients having a poor outcome at one-year follow up. Most patients died from systemic complications linked to their ICU stay, or treatment withdrawal for ethical purposes, while in the meantime, SE had resolved in half of them. However, half of long-term survivors recovered satisfactorily over time, though we did not identify any robust factor of good outcome. Even though randomized controlled studies may be extremely difficult to conduct in such a rare disease, the present findings urge for the need at least for large observational prospective studies.

## Key messages

- Prolonged refractory status epilepticus leads to high mortality
- Most patients die from systemic complications more related to their management in the intensive care unit than to brain damage
- Half of long-term survivors have a good outcome

## Abbreviations

AED: antiepileptic drugs
EEG: electroencephalogram
GA: general anesthesia
mRS: modified Rankin scale
PRSE: prolonged refractory status epilepticus
SE: status epilepticus
